# Differential role of M cells in enteroid infection by *Mycobacterium avium* subsp. *paratuberculosis* and *Salmonella enterica* serovar Typhimurium

**DOI:** 10.3389/fcimb.2024.1416537

**Published:** 2024-07-08

**Authors:** Omar A. Alfituri, Rosemary Blake, Kirsty Jensen, Neil A. Mabbott, Jayne Hope, Joanne M. Stevens

**Affiliations:** The Roslin Institute & Royal (Dick) School of Veterinary Studies, University of Edinburgh, Midlothian, United Kingdom

**Keywords:** *Mycobacterium avium* subsp. paratuberculosis, MAP, M cells, enteroids, mucosal immunity, RANKL, Johne’s disease

## Abstract

Infection of ruminants such as cattle with *Mycobacterium avium* subsp*. paratuberculosis* (MAP) causes Johne’s disease, a disease characterized by chronic inflammation of the small intestine and diarrhoea. Infection with MAP is acquired via the faecal-to-oral route and the pathogen initially invades the epithelial lining of the small intestine. In this study we used an *in vitro* 3D mouse enteroid model to determine the influence of M cells in infection of the gut epithelia by MAP, in comparison with another bacterial intestinal pathogen of veterinary importance, *Salmonella enterica* serovar Typhimurium. The differentiation of M cells in the enteroid cultures was induced by stimulation with the cytokine receptor activator of nuclear factor-κB ligand (RANKL), and the effects on MAP and *Salmonella* uptake and intracellular survival were determined. The presence of M cells in the cultures correlated with increased uptake and intracellular survival of *Salmonella*, but had no effect on MAP. Interestingly neither pathogen was observed to preferentially accumulate within GP2-positive M cells.

## Introduction

M cells are considered to play an important role in sampling the intestinal lumen for antigens and in inducing mucosal immunity against potential intestinal pathogens (Mabbott et al., 2013; Allaire et al., 2018; Dillon and Lo, 2019). These cells allow for the transcytosis of antigens to the Peyer’s patch where they are transmitted to macrophages and dendritic cells that are present in the Sub-epithelial dome (SED). As such, several pathogens have been shown to target M cells for their invasion and hijack the mucosal immune system. For example, the bacterial intestinal pathogen *Salmonella enterica* serovar Typhimurium (*Salmonella* Typhimurium) (Jepson and Clark, 2001) induces the differentiation of enterocytes in the FAE into M cells which increases bacterial invasion and colonization of the gut (Tahoun et al., 2012). The bacterium utilises the type III effector protein, SopB to activate the Wnt/β-catenin signalling pathway. This in turn leads to the induction of RANKL and the RANK receptor, allowing for the differentiation of FAE enterocytes into M cells (Tahoun et al., 2012). *In vivo* studies showed that mouse guts infected with SopB-deficient *S.* Typhimurium did not exhibit the same significant increase in GP2-expressing M cells as mice infected with WT bacteria. Further studies have also indicated the use of adhesion complexes in the bacterial attachment and entry into host cells (Shi and Casanova, 2006; Ly and Casanova, 2007). The bacterium uses focal adhesion kinase (FAK) and scaffolding proteins for its entry. In cells deficient in FAK, *Salmonella* invasion is significantly decreased. FAK has also been shown to play a role in the invasion of host cells during *Yersinia* and *Staphylococcus* infections (Eitel et al., 2005; Josse et al., 2017). However, there are several examples of bacterial invasion of host cells involving components of the host extracellular matrix (ECM); namely fibrinogen and integrins (Scibelli et al., 2007; Hoffmann et al., 2011; Josse et al., 2017). For example, *S.* Typhimurium uses fimbriae to attach to M cells and the bacterial protein ShdA to bind host fibronectin and collagen I for colonising the intestines (Kingsley et al., 2004; Rehman et al., 2019).

Another enteric pathogen, *Shigella flexneri*, has been shown to take advantage of M cells during its invasion of the gut epithelium (Ranganathan et al., 2019). Experiments using a human enteroid model showed that when M cell induction occurred, bacterial invasion increased by 10-fold. M cells have also been shown to play a pivotal role in prion pathogenesis, as orally infected mice that were treated with anti-RANKL antibodies exhibited a decreased prion load in the gut and blocked neuroinvasion from the intestines (Donaldson et al., 2012).

*Mycobacterium avium* subsp. *paratuberculosis* (MAP) is a member of the mycobacteriaceae family and is closely related to the non-tuberculosis *M. avium*. MAP is an acid-fast bacillus that causes Johne’s disease, or paratuberculosis, in ruminants. Johne’s disease is a serious infectious disease of the small intestine that causes significant health and production issues in cattle populations worldwide. As such, the impact from Johne’s disease has led to major economic losses in the dairy industry, due to significant reductions in milk yield, poor animal health and herd culling (Idris et al., 2021; Ssekitoleko et al., 2021). MAP transmission occurs via the faecal-to-oral route and infection occurs predominantly in calves within the first days or weeks of life. Infected cattle not only shed MAP in faecal matter, but also in colostrum and milk (Harris and Barletta, 2001; Health W.O.F.A, 2012). Following ingestion, the MAP bacterium initially infects the host via the epithelium of the small intestine. Once the MAP bacterium has crossed the gut epithelium it establishes infection in macrophages and dendritic cells where it can persist long-term and replicate (Rees et al., 2020). How MAP initially infects and crosses the gut epithelium is uncertain. M cells have also been implicated as potential sites of initial MAP uptake from the gut lumen into Peyer’s patches. Under experimental infections of animal hosts, MAP has been visualised within intestinal M cells of bovine calves, goats and mice (Momotani et al., 1988; Sigur-Dardóttir et al., 2001; Bermudez et al., 2010). The interaction of MAP with M cells has been postulated to involve a bacterial adhesin known as Fibronectin Attachment Protein (FAP-P), which mediates attachment to integrins expressed on the surface of M cells through a Fibronectin bridge (Secott et al., 2004). However, it has also been suggested that MAP can cross the intestinal epithelia independently of M cells (Sigurdardóttir et al., 2005) via enterocytes (Bermudez et al., 2010), or goblet cells (Schleig et al., 2005).

The development of 3D intestinal organoid cell cultures (termed enteroids) has enabled host-pathogen interactions in the intestinal epithelium to be studied at the cellular and molecular level (Clevers, 2016; Bartfeld and Clevers, 2017; Hamilton et al., 2018). Enteroids have the distinct advantage over monocultures of primary cells or cell lines in that they contain most of the cell types present in the intestinal epithelium *in vivo*. The epithelial cells within these enteroid cultures differentiate from Lgr5^+^ intestinal crypt stem cells. Although M cells are not present in these enteroid cultures in the steady state, their differentiation can be induced by stimulation with the cytokine receptor activator of nuclear factor-κB ligand (RANKL). RANKL stimulation of epithelial cells induces a suite of gene expression that includes the transcription factor Spi-B, which is required for the differentiation and maturation of these cells into functional M cells. At the time of writing induction of M cells in bovine enteroid cultures was not possible, due to the lack of biologically active bovine RANKL. Therefore, in the current study we used murine small intestinal enteroids to determine whether an increased abundance of M cells in the intestinal epithelium would increase the ability of MAP to infect the enteroids. Although mice are not the natural hosts of MAP, previous research has involved their use to study the early host-pathogen interactions (Rosseels et al., 2006; Bermudez et al., 2010). Here we show that, whereas an increased abundance of M cells increased the uptake of *S.* Typhimurium into the enteroids, the uptake of MAP was unaffected. Our data suggest that the initial uptake and transport of MAP across the small intestinal epithelium occurs independently of M cells.

## Materials and methods

### Isolation of intestinal crypts and enteroid culture

Healthy female C57BL/6 mice (6-8 weeks old) were sacrificed under the authority of a UK Home Office Project License, in accordance with the regulations of the UK Home Office “Animals (scientific procedures) Act 1986’’. All samples were taken with ethical approval from the Veterinary Ethics and Review Committee at the Royal (Dick) School of Veterinary Studies in line with the Animal Research: Reporting of *In Vivo* Experiments (ARRIVE) Guidelines (Percie du Sert et al., 2020). Approximately 20 cm of small intestine was dissected and crypts isolated as previously described (Sato et al., 2009). The intestinal crypts were resuspended in IntestiCult medium (StemCell Technologies) at approximately 4x10^3^ crypts/mL. The crypts were then mixed in Matrigel matrix (Corning, UK) at 1:1, and 50 µL drops were plated on pre-heated 24-well plates. After allowing the matrigel domes to set, 650 µL of pre-heated IntestiCult was added to each well and the enteroids incubated at 37°C with 5% CO_2_. The IntestiCult medium was replenished every 2 days and enteroids were passaged after 7 days. To assess RANKL stimulation, enteroids were treated with 50 ng/mL murine RANKL (Biolegend), where indicated.

### Bacterial culture

Previously frozen aliquots of C type MAP strain C49 WT (Mathie et al., 2020) and MAP C49 pWES4 (GFP+) strains were resuscitated by incubation in a shaking orbital incubator at 37°C overnight the day prior to the experiment. For enteroid infection, aliquots of MAP were centrifuged at 16,000 x g for 2 minutes and the supernatant removed. The bacterial pellets were then resuspended to 1.5 mL 7H9 medium. Prior to use, the MAP bacteria were then passed through a 30G needle 10 times. For strains of MAP that were constitutively expressing GFP, bacteria were cultured in 7H9 growth medium with 50 µg/mL kanamycin.

*Salmonella enterica* serovar Typhimurium ST4/74 was cultured in Luria-Bertani (LB) broth in an orbital shaking incubator at 37°C. Overnight cultures were diluted in LB broth prior to infection of enteroids. For strains of *S*. Typhimurium that were constitutively expressing GFP, bacteria were cultured in LB supplemented with 100 µg/mL ampicillin. To ascertain whether RANKL itself had any effect on the replication of *S.* Typhimurium, replication assays were performed whereby 1x10^6^ bacteria was cultured with or without 50 ng/mL murine RANKL, at 37°C for 1, 4, 8, and 24 hours. Following each time point, samples were plated on LB agar plates and CFU measurements were determined.

### Bacterial infection of enteroids

The enteroids were infected by disrupting the 3D enteroid structures to expose the luminal section and the bacteria were incubated with these enteroid segments. The disruption process follows the routine enteroid passage protocol, as described above. The IntestiCult medium was removed and replaced with 650 µL PBS, and the enteroids were resuspended and transferred into tubes. The suspended enteroid fragments were centrifuged at 40 x g for 2 minutes at 4°C and the supernatants carefully removed to avoid disrupting the cell pellet. The number of enteroid fragments were counted via microscopy to standardize for each experiment. The enteroid pellets were resuspended in the appropriate volume of bacteria suspension and incubated for 1 hour at 37°C at 5% CO_2_, with the aim of achieving an MOI of 100 MAP per enteroid fragment or an MOI of 10 *Salmonella* per enteroid fragment. After the 1 hour incubation, the enteroid: bacteria suspensions were washed twice in PBS to remove non-adherent bacteria by centrifugation at 40 x g for 2 minutes and removal of the supernatant. The one hour samples were collected for analysis, and duplicate suspensions for additional time point analysis were suspended in 50 µL Matrigel and plated onto pre-heated 24-well plates. The Matrigel domes were allowed to set for 30 minutes at 37°C before 650 µL of IntestiCult medium was added to each well. These infected enteroids were then incubated at 37°C, 5% CO_2_ for 24 and 72 hours. Following incubation, all the enteroids were processed for RNA, gDNA, colony forming unit (CFU) measurement or confocal microscopy analysis, as described below. For experiments including *Salmonella*, gentamicin (50 μg/mL) was included in the medium at time points after the one hour initial incubation with cells.

### Bacterial enumeration from infected enteroids

To ascertain the number of MAP following enteroid infection, gDNA was collected from each sample. The supernatant from each well was transferred to 15 mL falcon tubes, and the Matrigel domes were twice washed with 1 mL PBS, adding each supernatant to the relevant tube. The cells were trypsinised with 500 µL TrypLE Express and incubated at 37°C for 10 minutes. Following incubation, 1 mL DMEM F12 (10% FBS) was added to the wells and the cell suspensions were transferred to the relevant falcon tubes. The DMEM F12 wash was repeated. Cells were then centrifuged for 10 minutes at 1,800 x g. Supernatants were discarded and cell pellets were collected. gDNA was harvested using the Qiagen DNeasy Blood and Tissue extraction kit following the manufacturer's instructions, and gDNA was stored at -70°C until required. Each cell pellet was resuspended in 180 µL enzymatic lysis buffer (40 mg lysozyme/mL buffer) and incubated overnight at 37°C. Following incubation, 25 µL proteinase K and 200 µL buffer AL was added and samples mixed thoroughly. Samples were incubated for 2 hours at 56°C. MAP was then quantified by qPCR with primers designed to amplify the F57 sequence (Blake et al., 2022), a single copy gene unique to MAP. The qPCR was performed with SYBR green Supermix (Quantabio) with the appropriate negative controls to confirm the absence of contamination. Gene expression was quantified by delta delta CT method.

To quantify the number of *S.* Typhimurium following enteroid infection, enteroids were recovered and mechanically disrupted. Cells were lysed in 200 µL 0.1% v/v Triton X-100, serial dilutions prepared, plated onto agar plates and incubated for 19 hours at 37°C. Colonies were counted and the values used to calculate the numbers of viable bacteria present in each sample.

### Immunofluorescent staining and imaging of enteroids

To detect M cells in enteroid cultures, enteroids were fixed in 4% paraformaldehyde (PFA) and then immunostained with rat anti-mouse Glycoprotein 2 (GP2) monoclonal antibody (MBL International, USA). Samples were then stained with either Alexa Fluor 488-conjugated anti-rat IgG antibody or Alexa Fluor 647-conjugated anti-rat IgG antibody (Thermo Fisher Scientific). Where indicated, samples were stained with Alexa Fluor 647-conjugated phalloidin to detect F-actin (Thermo Fisher Scientific). DAPI was used to detect cell nuclei. Sections were mounted in fluorescent mounting medium (DAKO, Stockport, UK) prior to imaging on a Zeiss LSM 880 Airyscan/Fastscan confocal microscope (Zeiss, UK). For detecting bacterial invasion, GFP^+^ MAP and GFP^+^ *S.* Typhimurium ST4/74 were used.

### Real-time quantitative PCR analysis

For RNA extraction from enteroid cultures, the Trizol RNA extraction protocol was followed (Ambion). The SuperScript IV VILO Master Mix with ezDNase Enzyme was then used to remove genomic DNA and synthesize cDNA (Thermo Fisher Scientific). The PCRs were performed using the primers listed in **Table 1**, the Platinum SYBR Green qPCR SuperMix-UDG kit (Thermo Fisher Scientific) and Bio-Rad thermocycler. Gene expression was quantified by Delta Delta CT method and normalized to RPL19.

**Table 1:** Primers used for qPCRs.

Gene	Forward	Reverse
**RPL19**	GAAGGTCAAAGGGAATGTGTTCA	CCTTGTCTGCCTTCAGCTTGT
**GP2**	GATACTGCACAGACCCCTCCA	GCAGTTCCGGTCATTGAGGTA
**CCL20**	CGACTGTTGCCTCTCGTACA	AGCCCTTTTCACCCAGTTCT
**SpiB**	AGCGCATGACGTATCAGAAGC	GGAATCCTATACACGGCACAGG
**SOX8**	TCCGTTGCTCTCCGGTTT	GCCCATTCTCTCCTTTGTCCT
**F57**	GACTGGTAGACGCCCATTTC	GCTTAGTTCGCCGCTTGA

### Statistical analyses

Data are presented as the mean ± SD. Unless indicated otherwise, statistical differences between groups were compared by ANOVA using GraphPad Prism v9 (GraphPad software, USA). Data was normalized using D’Agostino & Pearson, Shapiro-Wilk and Kolmogorov-Smirnov normality tests. Data that did not pass the normality test were analyzed using appropriate non-parametric tests (specifically by Kruskal-Wallis test). P values < 0.05 were accepted as significant.

## Results

### RANKL-stimulation induces expression of M cell-related genes in murine enteroids

**Figure 1:**
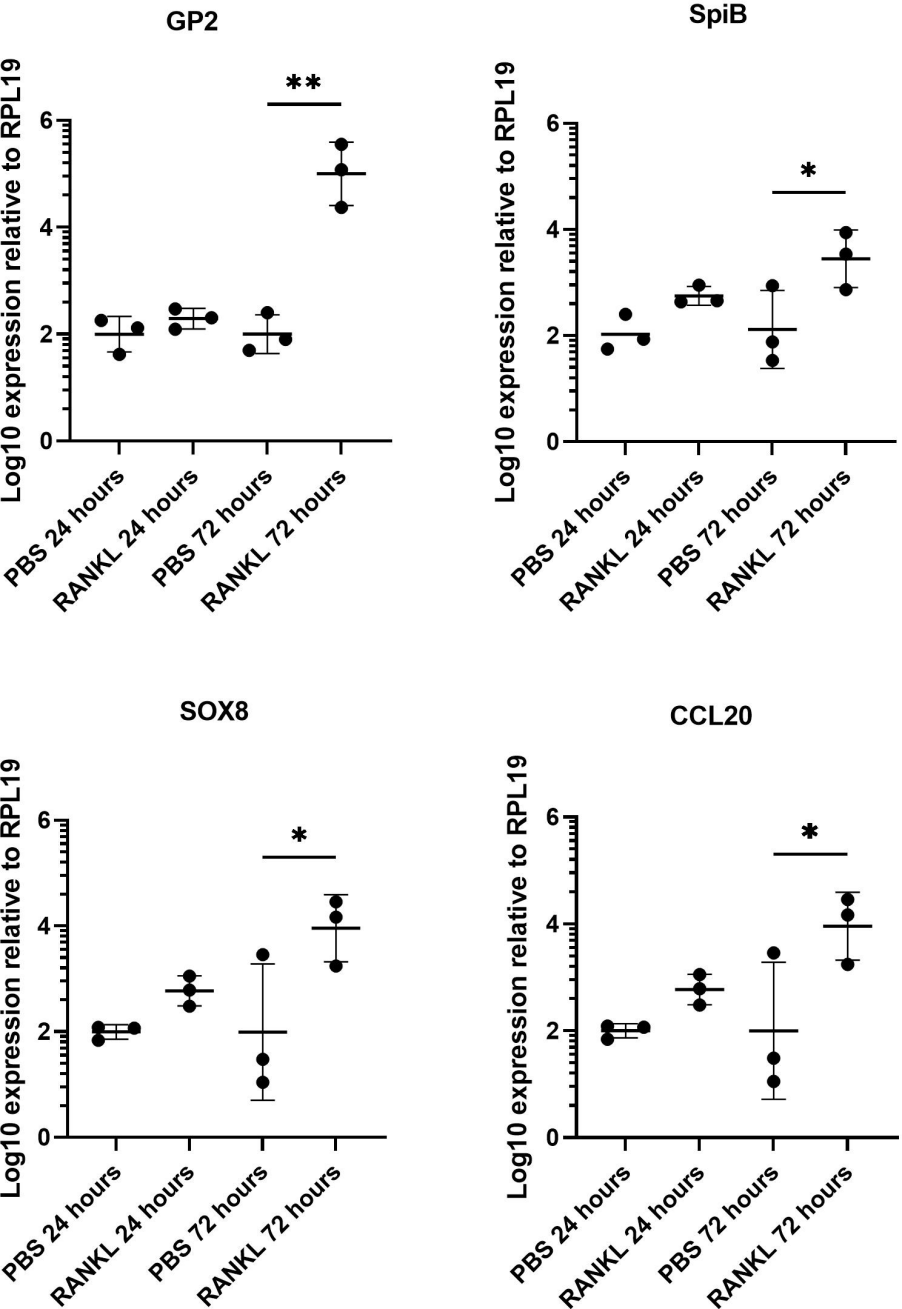
RANKL treatment upregulates M cell gene markers in murine enteroids. Murine enteroids were treated with RANKL (50 ng/mL) or PBS (control) and the expression of *GP2*, *SpiB*, *Sox8*, and *Ccl20* was compared by RT-qPCR 24 or 72 hours later. Each point represents the mean from triplicate wells, and the horizontal bar represents the mean ± SD. All experiments were repeated three times on different days (n = 3). Statistical differences were determined by ANOVA. *P < 0.05; **P < 0.01.

We first confirmed the effect of RANKL on M cell-differentiation in the murine enteroid cultures. RANKL stimulation of epithelial cells stimulates a programme of gene expression including the transcription factor Spi-B and Sox8 (Sato et al., 2013; Kimura et al., 2019) that ultimately leads to cell differentiation and maturation into M cells. Enteroids were stimulated with 50 ng/mL of RANKL for three consecutive days (medium alone was used as a control), and effects on the expression of M cell-related genes were determined by qRT-PCR. As anticipated from the study of Kanaya and colleagues (Kanaya et al., 2012), expression of mRNA encoding the mature M cell marker glycoprotein 2 (*Gp2*) was significantly elevated 400-fold compared to controls by 72 hours after RANKL-stimulation (**Figure 1**) (P=0.0017). The expression of *Spib* and *Sox8* mRNA was induced in the enteroids by 72 hours after RANKL-stimulation (**Figure 1**). The chemokine CCL20 is expressed by M cells and throughout the follicle-associated epithelium (FAE) covering the gut-associated lymphoid tissues, stimulating the recruitment of CCR6-expressing leukocytes towards the FAE (Ebisawa et al., 2011). Expression of *Ccl20* mRNA was significantly increased 4-fold in the enteroid cultures by 72 hours after RANKL-stimulation (**Figure 1**) (P=0.0341).

**Figure 2:**
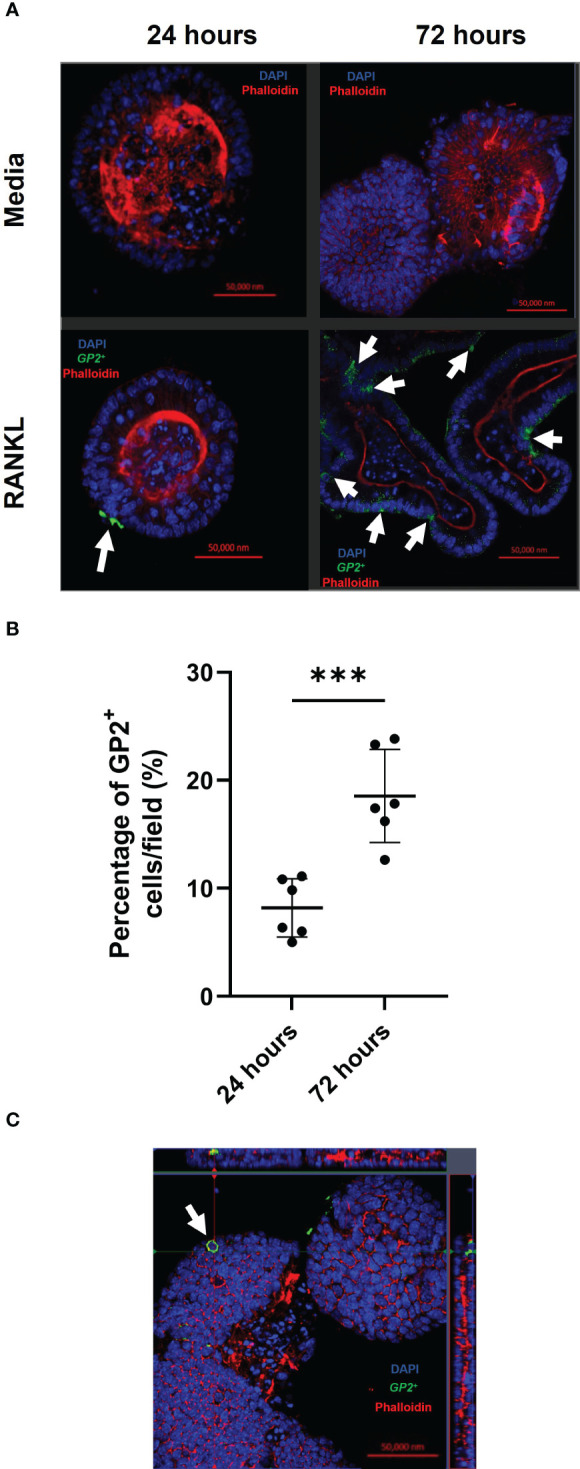
RANKL treatment enhances M cell differentiation in murine enteroids. **(A)** Confocal images of murine enteroids 24 hours following PBS treatment (control) (top panels). Immunostaining of GP2^+^ M cells (green), nuclei (blue) and F-actin (red) in murine enteroids, 24 hours following RANKL treatment (bottom panels). White arrows indicate the M cells. Scale bar 50 μm. **(B)** The percentage of GP2^+^ cells per field across the epithelium of enteroids after 24 and 72 hours post-RANKL stimulation. Cells were quantified using ImageJ software. Each point represents individual enteroids, and the horizontal bar represents the mean ± SD. n = 6. Statistical differences were determined by Student’s t-test. ***P < 0.001. **(C)** Confocal image of GP2^+^ M cells (green) in enteroids, 24 hours following RANKL treatment, displaying the characteristic “doughnut” appearance. Cell nuclei (blue), F-actin (red) (bottom panels).

Concurrent with the gene expression data, abundant GP2^+^ M cells were detected in the enteroid cultures 24 and 72 hours after RANKL-stimulation (**Figure 2A**). The top panels show enteroids cultured without RANKL, while the bottom panels show enteroids cultured with RANKL after 24 and 72 hours. These images show clear induction of GP2^+^ M cells (green cells indicated by white arrows). To quantify the levels of GP2^+^ M cell induction, ImageJ software was used to calculate the percentage of GP2^+^ cells per field across the epithelium of enteroids after 24 and 72 hours post-RANKL stimulation (**Figure 2B**). These data showed significantly higher GP2^+^ M cell numbers by 72 hours post-RANKL stimulation when compared with 24 hours post-RANKL stimulation (P=0.0006). The confocal imaging shows that the GP2 expression in the M cells exhibit a “doughnut-like” structure around the cell (**Figure 2C**). Together, these data confirm that M cell differentiation was induced in the murine enteroids by RANKL-stimulation.

### RANKL-stimulation of enteroids increases susceptibility to *Salmonella* Typhimurium infection

**Figure 3:**
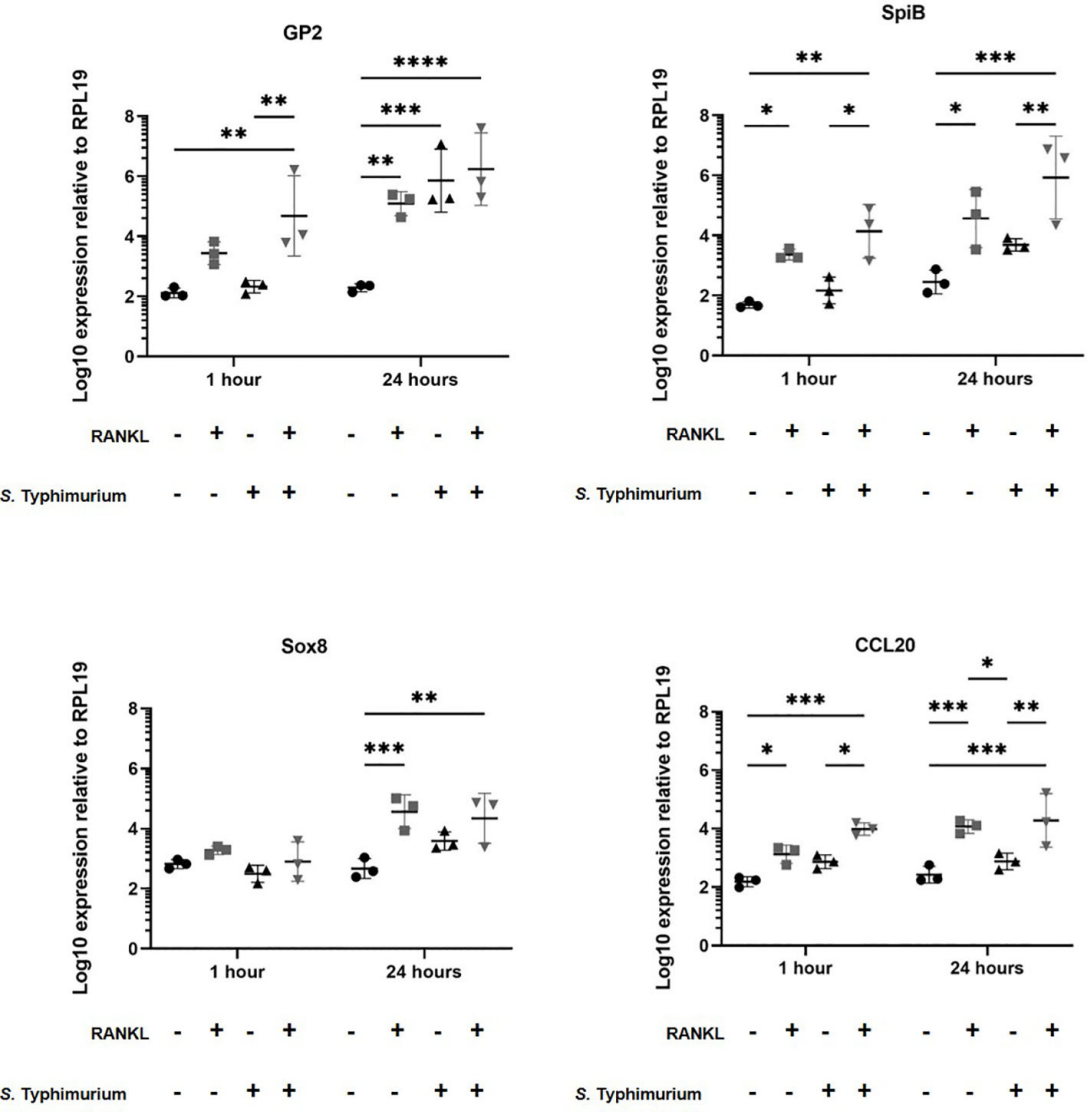
*Salmonella* Typhimurium infection enhances M cell differentiation in RANKL treated murine enteroids. Murine enteroids were treated with RANKL (50 ng/mL) or PBS (control) for 72 hours before being infected with *S*. Typhimurium at an MOI of 10 bacteria per enteroid fragment. Uninfected enteroids were used as controls. The expression of *GP2*, *SpiB*, *Sox8*, and *Ccl20* was compared by RT-qPCR 1 and 24 hours post-infection. n = 3. All experiments were repeated three times on different days (n=3). Uninfected + PBS (circle); uninfected + RANKL (square); Salmonella + PBS (upwards triangle); Salmonella + RANKL (downwards triangle). Statistical differences were determined by ANOVA. Each point represents the mean from triplicate wells, and the horizontal bar represents the mean ± SD. *P < 0.05; **P < 0.01; ***P < 0.001; ****P < 0.0001.

Certain pathogenic bacteria such as *Salmonella enterica* serovar Typhimurium can bind to GP2 on the surface of M cells and use this to cross the gut epithelium and enter host tissues (Hase et al., 2009). We next determined whether the RANKL-mediated increase in M cell-abundance would enhance the infection of enteroids with *S.* Typhimurium bacteria. The apical surface of the M cells and other epithelial cell subsets in the enteroids orientates towards the closed lumenal surface. Therefore, in order to establish infection via the lumenal apical surface, the enteroids were mechanically disrupted and *S.* Typhimurium bacteria were added before the dissociated enteroid crypt domains had re-sealed. Enteroids were infected at an MOI of 10 bacteria per enteroid fragment, for a maximum of 24 hours. Higher MOIs or longer infection periods were hampered by the cytotoxicity that the bacteria exerted on the viability of the cells. The enteroids were then washed to remove non-adherent bacteria, subsequently cultured in Matrigel domes in the presence of gentamicin to kill any extracellular bacteria, and sampled at intervals afterwards. In the first experiment, we compared gene expression in enteroids with and without a 72-hour pretreatment with RANKL, with or without infection with *S.* Typhimurium. Consistent with the data presented in **Figure 1**, and in the absence of infection, the M cell-related genes *Gp2*, *Spib*, *Sox8* and *Ccl20* were significantly increased in their expression levels (**Figure 3**, compare circles and boxes in plots). In the M-cell deficient enteroids (- RANKL), the presence of *Salmonella* stimulated an increased expression of *Gp2* by 24 hours post-infection (**Figure 3**, compare circles and triangles in the plots). This is consistent with the demonstration that *S.* Typhimurium itself can enhance the differentiation of enterocytes in the FAE into M cells (Tahoun et al., 2012). In M cell +ve (RANKL stimulated) enteroids, infection with *Salmonella* led to a further increase in expression of *Gp2* (at one hour post-infection), *SpiB* and *Ccl20* (at both 1 and 24 hours post-infection), compared to the uninfected RANKL stimulated enteroids (**Figure 3**, compare triangles with inverted triangles in plots).

**Figure 4:**
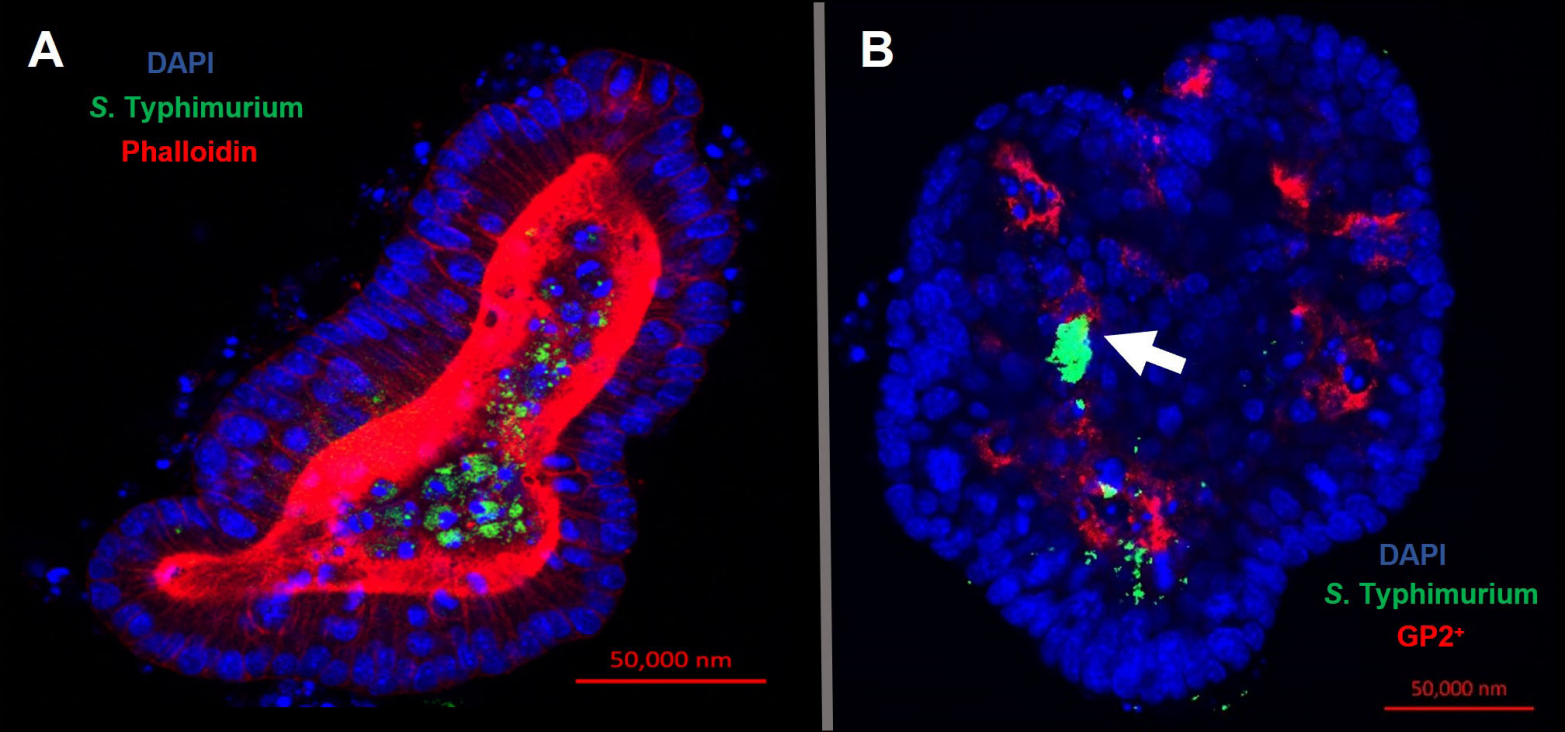
Intracellular *S*. Typhimurium does not accumulate in GP2-positive M cells. Confocal microscope images of murine enteroids infected with GFP^+^ *S*. Typhimurium for 24 hours. **(A)** *S.* Typhimurium (green), cell nuclei (blue), F-actin (red). **(B)** *S.* Typhimurium (green), cell nuclei (blue), GP2 (red). Scale bar 50 μm.

**Figure 5:**
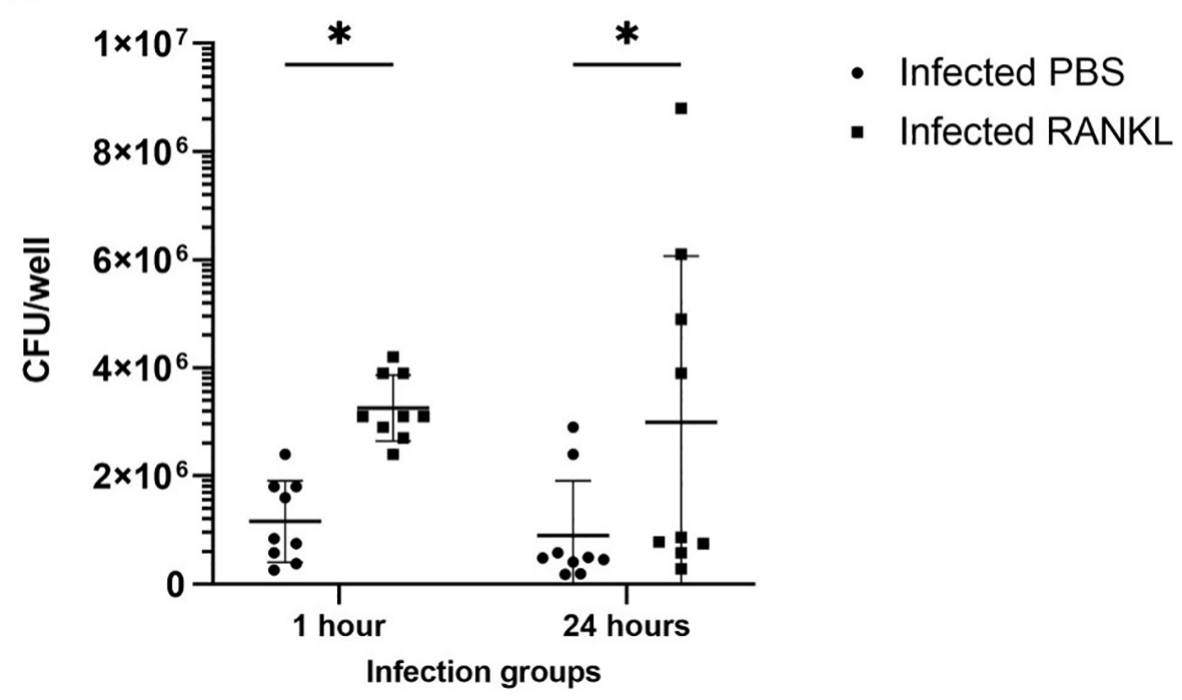
Invasion and survival of *S*. Typhimurium in enteroid cultures. Bacteria were enumerated after infection of enteroids with and without RANKL stimulation, at 1 and 24 hours post-infection. All experiments were repeated three times on different days (n=3), each with 3 technical replicates for each condition. Data is expressed as the mean ± SD. Statistical differences were determined by ANOVA. *P < 0.05.

Confocal microscopy demonstrates the presence of intracellular *S*. Typhimurium in numerous cells of enteroids at 24 hours post-infection (**Figure 4A**). However, it is noteworthy that the bacteria did not preferentially accumulate in GP2-positive M cells (**Figure 4B**). To determine the fate of the bacteria in the enteroids pre-treated with or without RANKL, and over time, colony forming units (CFUs) were calculated to enumerate the numbers of viable bacteria. This analysis showed that the abundance of *S*. Typhimurium in the enteroid cultures were significantly greater when pre-stimulated with RANKL at both 1 hour (P = 0.0254) and 24 hour (P = 0.0256) post-infection, compared to the unstimulated controls (**Figure 5**). This difference is not due to any direct effect of the RANKL on replication of the bacterium (data not shown). This implies that a greater number of bacteria adhered to and entered the RANKL-stimulated enteroid cells during the one hour infection stage of the experiment, compared to the control enteroid cells. The numbers of bacteria over time did not change significantly, regardless of RANKL treatment. This is most likely indicative of a lack of bacterial replication in the infected cells. Other reports have suggested that M cells may act as a portal of entry into the gut epithelium, providing a protective niche in which the bacteria can survive (Tahoun et al., 2012). Whilst we have observed an increase in initial uptake of *Salmonella* in RANKL-treated M cell-containing enteroid cultures, these bacteria were not specifically localized within GP2-positive cells.

### RANKL-stimulation does not affect the susceptibility of enteroids to MAP infection

**Figure 6:**
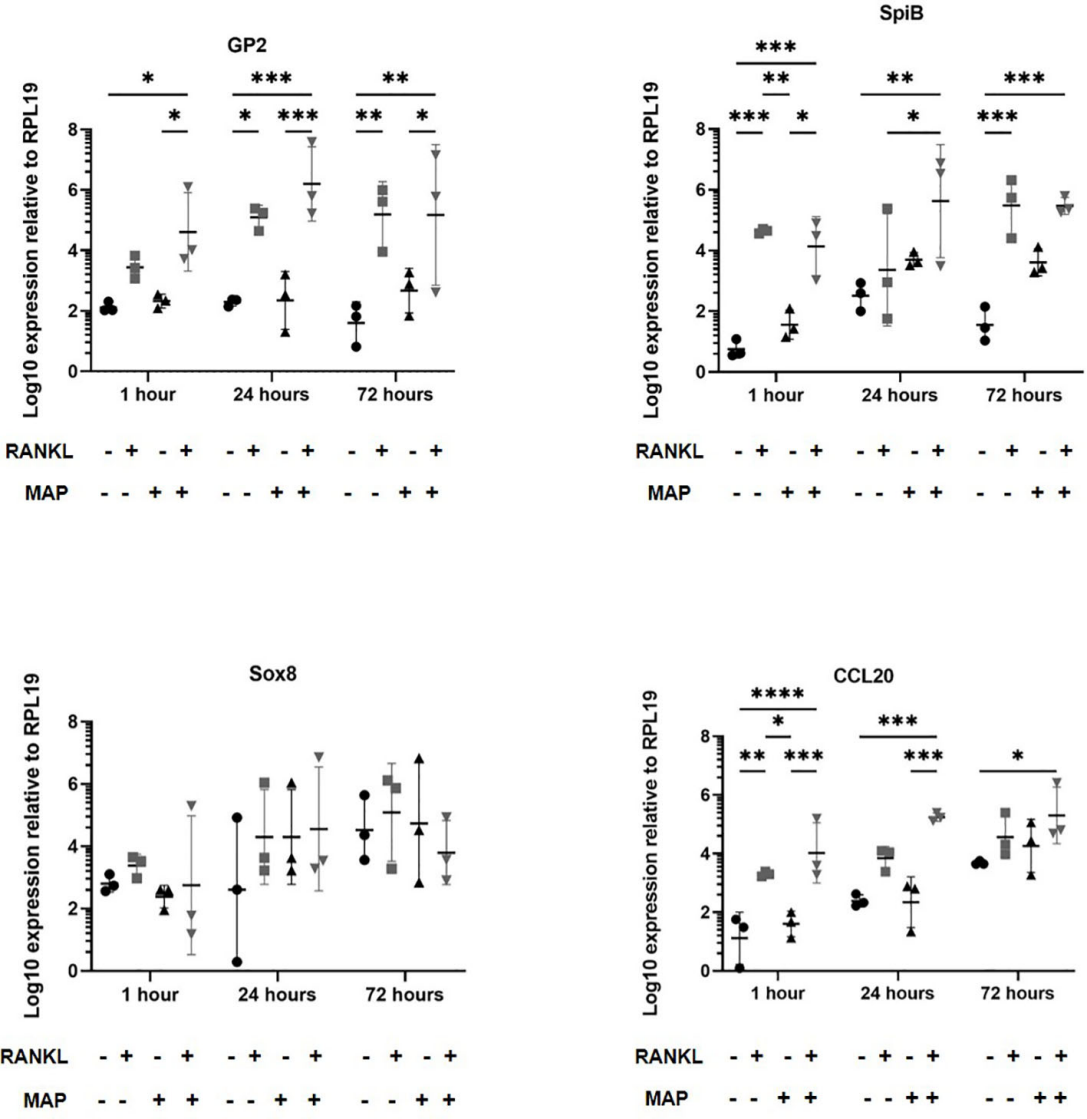
MAP-infection of murine enteroids does not affect M cell differentiation gene marker expression. Murine enteroids were treated with RANKL (50 ng/mL) or PBS (control) for 72 hours before being infected with MAP at an MOI of 100 bacteria per enteroid fragment. Uninfected enteroids were used as controls. The expression of *GP2*, *SpiB*, *Sox8*, and *Ccl20* was compared by RT-qPCR 1, 24, or 72 hours later. All experiments were repeated three times on different days (n = 3). Each point represents the mean from triplicate wells, and the horizontal bar represents the mean ± SD. Uninfected + PBS (circle); uninfected + RANKL (square); MAP + PBS (upwards triangle); MAP + RANKL (downwards triangle). Statistical differences were determined by ANOVA. *P < 0.05, **P < 0.01; ***P < 0.001; ****P < 0.0001.

We next determined whether RANKL-stimulation similarly enhanced the infection of enteroids infected with *Mycobacterium avium* subsp. *paratuberculosis* (MAP), another enteric bacterial pathogen whose pathogenesis has been associated with M cells. Enteroid cultures were prepared and dissociated as with the *S*. Typhimurium. The enteroids were mechanically disrupted and exposed to MAP bacteria before the dissociated enteroid crypt domains had re-sealed. The enteroids were then washed to remove non-adherent bacteria, subsequently cultivated in Matrigel domes and sampled at intervals afterwards. In experiments involving MAP, an MOI of 100 bacteria per enteroid fragment and time points up to 72 hours post-infection were sampled. In our experience, MAP displays very little cytotoxic effect on mammalian cells even at high densities and over long periods of time. As described above, we first cultured enteroids with and without RANKL pre-treatment and in the presence or absence of MAP. Extracted RNA was gathered to perform qPCRs to determine whether RANKL stimulation induced different expression of the M cell-related genes *Gp2*, *Spib*, *Sox8* and *Ccl20* (**Figure 6**). These data showed that RANKL stimulation prior to MAP infection induced significantly increased levels of *Gp2*, *Spib*, and *Ccl20* up to 72 hours post-infection (consistent with the data shown in **Figures 1**, **3**). However, in contrast to our findings upon *S*. Typhimurium infection, and despite infecting the cells with 10 times more bacteria, MAP alone did not induce increased expression of *Gp2*, or any of the other M cell differentiation gene markers tested. This may reflect the significant difference in the cell envelope compositions of these two bacteria.

**Figure 7:**
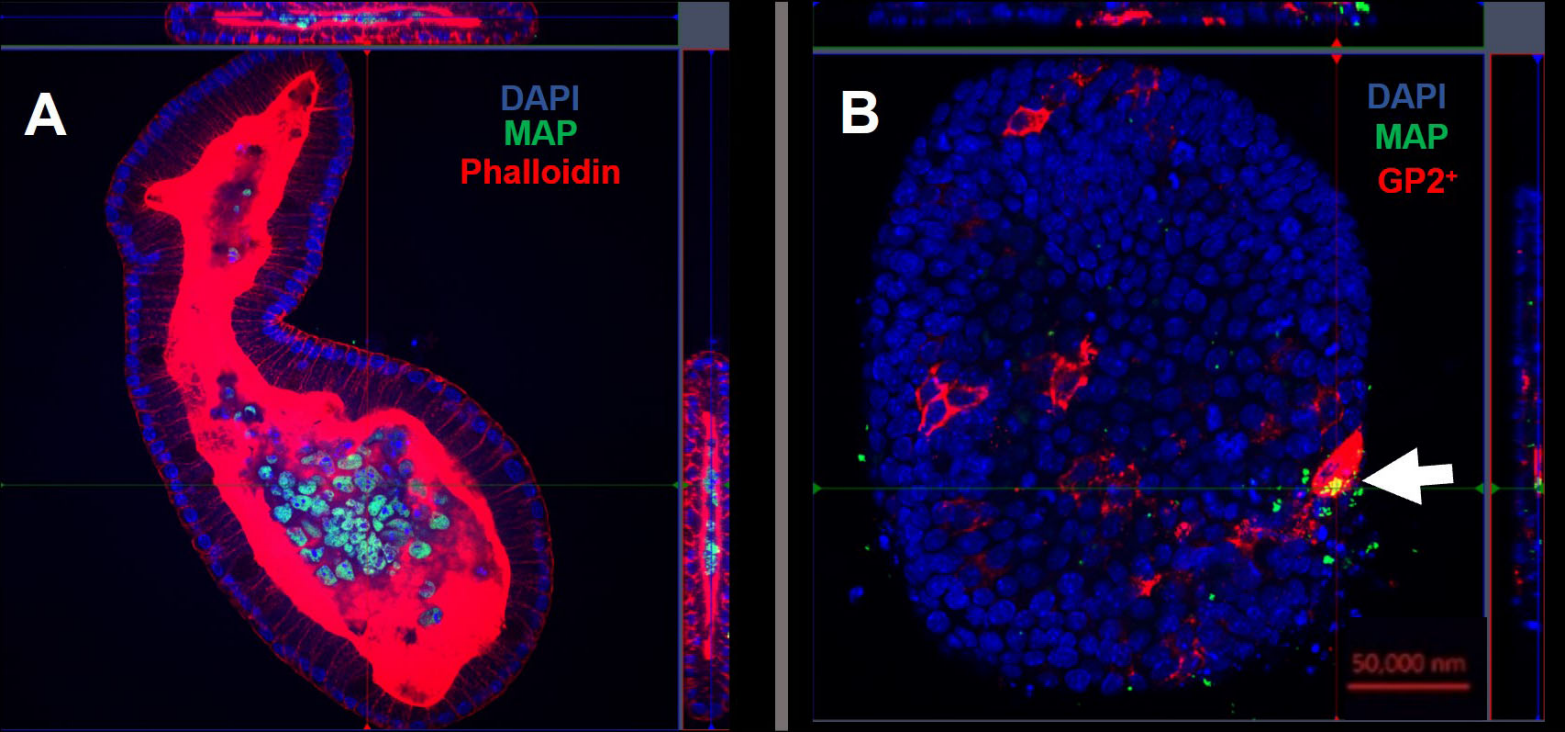
Intracellular MAP does not accumulate in GP2-positive M cells. Confocal microscope images of murine enteroids infected with GFP+ MAP for 24 hours. **(A)** MAP (green), cell nuclei (blue), F-actin (red). **(B)** MAP (green), cell nuclei (blue), GP2 (red). Scale bar 50 μm.

**Figure 8:**
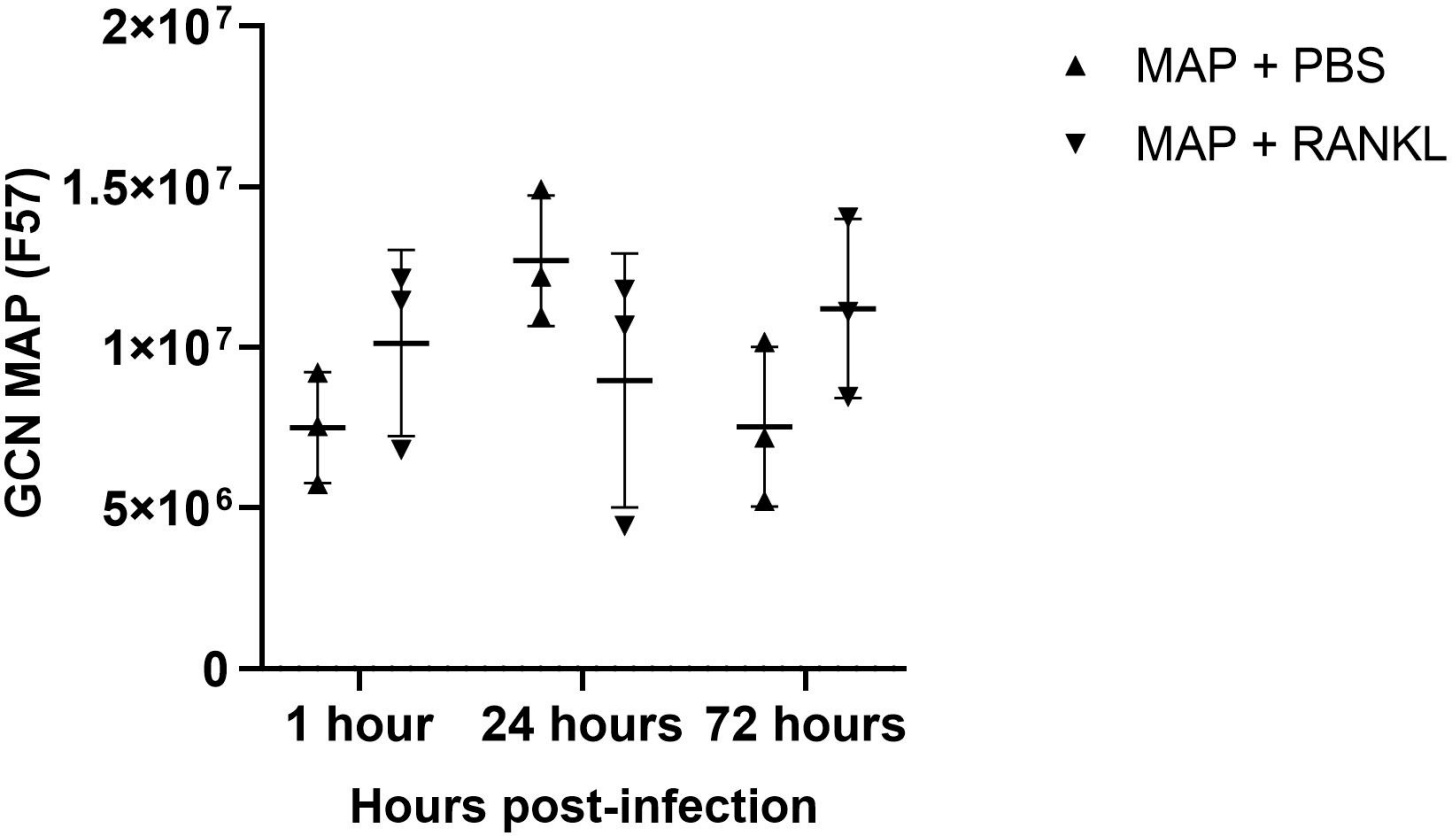
Enumeration of MAP genome copy number from infected enteroids treated with/without RANKL. Quantitative PCRs were performed to assess F57 gene copy number in infected murine enteroids treated with or without RANKL, at 1, 24 and 72 hours post-infection with MAP. All experiments were repeated three times on different days (n=3). Uninfected + PBS (circle); uninfected + RANKL (square); MAP + PBS (upwards triangle); MAP + RANKL (downwards triangle). n = 3. Each point represents the mean from triplicate wells, and the horizontal bar represents the mean ± SD. Statistical differences were determined by ANOVA.

Using confocal microscopy, we confirmed that the enteroid cells were infected and contained intracellular bacteria at 24 hours post-infection (**Figure 7A**). Similar to the findings with S. Typhimurium infection, MAP did not readily co-localize within GP2-positive M cells (**Figure 7B**). To determine the role of M cells in the uptake and survival of the bacteria within enteroid cells, we enumerated the bacteria by lysing infected cells and quantifying the number of bacterial genomes by qPCR. This method was chosen in consideration of the long incubation times required for recovery of MAP colonies on agar plates, and to overcome the common problem of underestimating actual viable bacterial counts because of the propensity of this bacterium to form clumps *in vitro*. However, we recognize that data from this method of enumeration represents the total number of both viable and dead bacteria present in a given sample. PCR quantification of the F57 sequence of MAP ascertained that the MAP genome copy numbers remained statistically similar between unstimulated and RANKL pre-stimulated enteroids, and did not significantly increase or decrease over time (**Figure 8**). The number of MAP bacteria up to 72 hours post-infection remained similar to the earlier time points, suggesting a lack of replication of the intracellular population of bacteria. Together, these data suggest that RANKL-mediated M cell-differentiation did not enhance the uptake of MAP in this *in vitro* model system of the small intestinal epithelium.

## Discussion

Several studies have stated the importance of M cells in the pathogenesis of the intestinal bacterial pathogens *Salmonella enterica* serovar Typhimurium (*Salmonella* Typhimurium) and *Mycobacterium avium* subsp. paratuberculosis (MAP). M cells have been described as the ‘portal of entry’ of these bacteria, resulting in transcytosis across Peyer’s patches and interaction with sub-epithelial lymphoid cells which assist in bacterial dissemination or bacterial survival within the host. In this study we hypothesized that both bacteria would co-localize with M cells in a murine enteroid model system, and that association with this cell type would lead to bacterial intracellular survival and potentially bacterial replication. Surprisingly, neither bacterium specifically co-localized within M cells, although the presence of M cells did lead to an increase in overall uptake and intracellular survival of *Salmonella* Typhimurium.

The close relative of MAP, *M. tuberculosis*, has been shown to utilise M cells in the lung as an alternative mechanism of entry and invasion of alveolar macrophages (Teitelbaum et al., 1999). In fact, studies in M cell-deficient mice have shown that *M. tuberculosis* dissemination to the draining lymph nodes and overall disease progression is significantly reduced (Nair et al., 2016; Bussi and Gutierrez, 2019). In addition, electron micrographs of *in vitro* monolayer models showed that *M. tuberculosis* can enter and translocate across M cells. Together our data suggest that although M cells have been shown to have important roles for other pathogens such as *Salmonella*, *Shigella* and prions, their increased abundance had no direct effect on MAP invasion in an enteroid system. Although, the caveat remains that we have studied MAP: M cell interactions in murine cells rather than using cells from a natural host. Bovine models may exhibit a different response, and we have previously shown that bovine enteroids provide a viable model for studying MAP infection and investigating the early stages of its pathogenesis (Blake et al., 2022). However, characterizing M cells in bovine enteroids remains an issue due to a lack of a specific cellular marker. With an appropriate bovine M cell marker, we could investigate bovine RANKL in a similar model to ascertain whether increased M cell numbers has an effect on MAP uptake.

Studies have shown that MAP is capable of binding ECM components along the gut epithelium. Research has shown that MAP can bind fibronectin via fibronectin attachment proteins (FAPs), and are activated in the intestines to do so in that specific pH environment (Middleton et al., 2000). These studies showed that when the FAP gene was absent, MAP adherence to the ECM was decreased. In addition, MAP treated with acid (pH range 3 to 10), allowed for greater binding of fibronectin (Secott et al., 2001). Blocking the fibronectin with binding peptides from the MAP FAP prevented fibronectin binding, suggesting MAP attachment is FAP dependent. M cells express high numbers of β1 integrins on their luminal surfaces and MAP may utilise its FAP and host fibronectin to enhance bacterial uptake in the gut (Secott et al., 2004). The invasion of M cells by MAP was found to be enhanced almost 3-fold when the bacteria was treated with fibronectin. Furthermore, blocking the fibronectin: integrin binding regions led to significantly reduced invasion of M cells by MAP of up to 75%. It is thought that MAP uses its FAP to bind fibronectin, and when in the small intestines, binds to M cells via their β1 integrins. Fibronectin bound to MAP will bind to the α5β1 integrin receptor complex to create a bridge of adherence for entry into the host cell (Secott et al., 2002). The sugar mannose (mannan) has previously been shown to be involved in bacterial adherence to host cell surfaces (Sharon et al., 1981). Studies of *Escherichia coli* has highlighted the role of mannose-binding lectins on the bacterial surface, which allows for host cell attachment and entry (Ofek and Beachey, 1978). Research has also shown that mannose-binding lectins amplify the uptake of *Staphylococcus aureus* and *E. coli* by Kupffer cells found in the liver (Ono et al., 2006). Similarly, MBLs increase opsonophagocytosis of *Salmonella enterica* serovars Typhimurium and Montevideo (Kuhlman et al., 1989; Devyatyarova-Johnson et al., 2000; Ulrich-Lynge et al., 2015). During *M. tuberculosis* infections, infected macrophages undergo apoptosis to induce an inflammatory immune response. However, experiments have shown that the bacteria induce apoptotic infected macrophages to be phagocytosed via mechanisms involving the mannose receptor (Garcia-Aguilar et al., 2016). Inhibiting the mannose receptor resulted in reduced phagocytosis of infected and apoptotic cells. Therefore, further studies are now required to ascertain whether opsonizing MAP with ECM proteins such as fibrinogen, or mannose, in the presence of increased M cells will affect bacterial uptake.

Johne’s disease remains a major economical strain on the cattle and dairy industries. The infection has a long sub-clinical period of around 2-4 years, in which fecal shedding and transmission can occur. The lack of clear clinical signs and diagnostic testing for detecting infectious animals also remains problematic. As such, a better understanding of the early entry mechanisms of MAP into the host intestine would be invaluable in designing novel strategies to impede infection, and hence transmission to other susceptible hosts.

## Data availability statement

The raw data supporting the conclusions of this article will be made available by the authors, without undue reservation.

## Author contributions

OA: Conceptualization, Data curation, Formal analysis, Investigation, Methodology, Writing – original draft, Writing – review & editing. RB: Methodology, Writing – review & editing. KJ: Investigation, Methodology, Writing – review & editing. NM: Conceptualization, Funding acquisition, Methodology, Supervision, Writing – review & editing. JH: Conceptualization, Funding acquisition, Methodology, Resources, Supervision, Writing – review & editing. JS: Conceptualization, Formal analysis, Funding acquisition, Methodology, Resources, Supervision, Writing – original draft, Writing – review & editing.
